# Caffeinated Drinks Intake, Late Chronotype, and Increased Body Mass Index among Medical Students in Chongqing, China: A Multiple Mediation Model

**DOI:** 10.3390/ijerph15081721

**Published:** 2018-08-10

**Authors:** Yangchang Zhang, Yang Xiong, Jia Dong, Tingting Guo, Xiaoman Tang, Yong Zhao

**Affiliations:** 1 Department of Public Health and Management, Chongqing Medical University, Chongqing 400016, China; 18702370226@163.com; 2 Department of Nursing, Chongqing Medical University, Chongqing 400016, China; 3 Department of the First Clinical Medicine, Chongqing Medical University, Chongqing 400016, China; cqmu_xy@163.com (Y.X.); kristydj@sina.com (J.D.); g500233@sina.com (T.G.); 18875211364m0@sina.cn (X.T.); 4 The Innovation Center for Social Risk Governance in Health, Chongqing Medical University, Chongqing 400016, China; 5 Research Center for Medicine and Social Development, Chongqing Medical University, Chongqing 400016, China

**Keywords:** medical students, chronotype, obesity, caffeinated drinks

## Abstract

*Background*: This paper investigates the problems regarding caffeinated drinks intake, late chronotype, and increased body mass index (BMI) among medical students at a Chinese university. *Methods*: This cross-sectional study was conducted in 2018 with 616 medical students from Chongqing Medical University in Chongqing, China, whose information were collected by a self-reported questionnaire that included four sections: Demographic characteristics; Caffeinated drinks intake and physical state; Morningness-Eveningness Questionnaire; Depression Anxiety Stress Scale 21. Multiple mediation analyses were conducted to test the impact of late chronotype on increased BMI through caffeinated drinks consumption through two models. *Results*: The significantly mediated effect of caffeinated drinks consumption was revealed (estimate: −0.01, SE = 0.01, 95% CI [−0.02, −0.01]), and which played a positive role in linking late chronotype (B = −0.01, SE = 0.01, *p* < 0.001) and increased BMI (B = 1.37, SE = 0.21, *p* < 0.01), but their significant association did not be found in reversed model. In addition, physical activity and inactivity times demonstrated significant indirect effects in the two models. *Conclusions*: Interventions should focus on reducing caffeinated drinks intake and sedentary behavior time, enhancing physical activity among medical students.

## 1. Introduction

Obesity is currently a major medical condition that has attracted considerable attention in the field of public health and is positively associated with the development of chronic diseases, such as type 2 diabetes, cardiovascular diseases, and sleep problems [1,2,3], and can even lead to cancer due to metabolic syndromes [4]. According to the survey form world health organization, a nearly 39% prevalence of overweight condition and 13% prevalence of obesity among adults were reported worldwide in 2016 [5]. In addition, the prevalence of obesity has consistently grown from 1993 to 2009, which has increased from 2.9% to 11.4% among males and from 8.5% to 27.8% among females in Chinese populations [6]. Therefore, identifying factors associated with Chinese populations in terms of overweight and obesity is critical for addressing this pressing public health issue.

Several reviews have reported that many kinds of physiological, environmental, and self-behavior factors can influence overweight and obesity [7,8,9]. Among these factors, personal life and behavior factors (e.g., physical activity, food intake) are becoming increasingly popular in weight regulation research [10,11]. However, the association between obesity and sleep has been overlooked, and many current studies have mainly focused on sleep duration and quality, and ignored the impact of endogenous biological rhythms. Poor biological rhythms may influence hormone metabolism, such as leptin, insulin, and cortisol, leading to obesity [12]. Additionally, different sleep-wake patterns exist in humans. For example, chronotype is considered a characteristic assessment mode that can describe individual sleep and activity time and is mainly divided into three types, namely, “morningness”, “intermediate” and “eveningness”. “Morningness” (“early larks”) refer to these people who prefer to maintain mental and physical alertness in the morning hours and feel in excellent condition. Eveningness (“night owls”) is the reverse, in that these people prefer to stay up late and get up at a later time [13]. Furthermore, the chronotype may potentially modify individual life behavior and eating time [14]. Therefore, chronotype could be hypothetically identified as independently associated with other behavioral risk factors about being overweight and obesity (e.g., alcohol, tobacco, and caffeine) [15].

As previously stated, late chronotype may be crucial in obesity prevalence research. However, the causation between late chronotype and stimulant consumption has not been well clarified in affecting weight change. The increased caffeinated drinks in the late chronotype group may provide a potential hypothesis for this issue. Some studies conducted in Brazil, England, and the US have already described that late chronotype is associated with the risk of additional caffeinated drinks intake [16,17,18], promoting beverage addiction and leading to weight gain. In recent years, caffeinated drinks have become more popular, especially in young groups, such as college students. Interestingly, problems on caffeinated drinks and late chronotype have been often reported together. The association between with late chronotype and caffeinated drinks consumption has been significantly revealed in the literature to experience a positive rise among college students according to cross-sectional study [18,19,20].

College students who are an extreme evening type may voluntarily shorten their hours of sleep in response to exams, to review lessons, or for entertainment and social contact purposes. Notably, additional caffeinated intake is acquired to maintain focus. Furthermore, medical students may be more affected by sleep disturbance compared with other majors because they experience intense academic pressure and spend additional years in school [21]. A previous study indicated that medical students have a high prevalence of poor sleep quality, which is associated with late chronotype and excessive daytime sleepiness [22]. Other factors have been identified, such as gender, age, residence, chronic health conditions, psychological distress and even college major, that may also affect the development of a late chronotype [20,22,23].

In addition, physical activity, including physical and psychological health, is known to be crucial for maintaining the stabilization of the human body [24]. The association between sedentary behavior and physical inactivity has been linked to overweight and obesity and this has attracted considerable attention in the field of public health [25]. Therefore, physical activity time is also a potential impact factor that we examined in this study.

Referring to previous studies, the effect of continuous caffeinated drinks consumption may play an underlying pathway role on weight regulation by mediating the relationship between chronotype and BMI. The aim of this study is to explore whether increased caffeinated drinks consumption can mediates the relationship between late chronotype and BMI in a sample of medical university students. In addition, we also investigated that the effect of physical activity time and physical inactivity time as other key mediators in mediation model.

## 2. Materials and Methods

### 2.1. Ethics Statement

The study protocol was approved by the Ethics Committee of Chongqing Medical University and was conducted in accordance with the Declaration of Helsinki (2016001).

### 2.2. Study Design and Sample

A total of 616 students were recruited from Chongqing Medical University in Chongqing, China. Seven classes from three departments of the school were randomly selected by cluster sampling and randomization to participate in the experiment. We contacted the class counselors and monitors to issue questionnaires before the formal survey. After a brief introduction to the questionnaire, the students filled it out separately and anonymously on the spot and the results were recorded by two trained researchers. Inclusion criterion was full-time undergraduates of the school, and exclusion criteria were participants who have a history of major diseases, chronic health condition, or mental trauma and those who submitted questionnaires with incomplete answers. Data were collected from November 2017 to April 2018. All participants provided written informed consent prior to the experiment.

### 2.3. Measures

#### 2.3.1. Demographic Variables

All participants provided complete questionnaire information, including gender (male/female), age (years), residence (campus/other places), and grades (freshman/sophomore/junior). BMI was obtained by filling in height and weight information, and calculation formula was weight (kg)/height (m^2^).

#### 2.3.2. Chronotype

The Morningness-Eveningness Questionnaire (MEQ) is a self-reported instrument developed to present individual sleep habits and circadian typology, which consisted of 19 questions associated with the participant’s sleeping onset and offset habits, preferred individual mental and physical activity, and alertness during the daytime; the MEQ has been adopted and applied for the Chinese population [26]. MEQ scores range from 16 to 86, and the high scores tend to show the strong features of morningness, and low scores are more inclined to show eveningness. Chronotypes were classified as morning types (59–86), intermediate types (42–58), and evening types (16–41) [27]. Cronbach’s α was 0.85 in this study.

#### 2.3.3. Physical Activity and Sedentary Behavior

Physical activity levels were assessed using the open-ended question: “How many hours or minutes for the past week do you usually spend on moderate activity, such as carrying packet on average working days and rest days?” Moreover, sedentary behavior information was acquired through the following question: “How many hours or minutes for the past week do you usually spend on physical inactivity, such as reading, surfing the Internet, watching TV, and studying on average working days and rest days?”

#### 2.3.4. Caffeinated Drinks Consumption

Participants were asked regarding their caffeinated drinks consumption during the past seven days: “How many bottles or tins of caffeinated drinks do you usually drinks on average per day?” caffeinated drinks included Red Bull, milk tea, herbal tea, soda water, coffee, and other caffeinated drinks, and the capacity of one bottle is approximately 500 mL (a tin is approximately 330 mL).

#### 2.3.5. Psychological Distress

The Depression Anxiety Stress Scale 21 (DASS-C21), which is a 21-item scale involving three self-report subscales revised to assess the participant’s mental condition including the states of depression, anxiety and stress [28]. The three subscales are as follows: (1) Stress scores are categorized as low (0–18) and moderate/extremely severe (19 and above); (2) Anxiety scores are categorized as low (0–9) and moderate/extremely severe (10 and above); (3) Depression scores are categorized as low (0–13) and moderate/extremely severe (14 and above). The DASS-C21 was already been applied to student groups and has shown excellent reliability and validity in Chinese populations [29,30]. In this pre-study, Cronbach’s α were 0.73, 0.76, and 0.88.

### 2.4. Primary and Reversed Model Constructed

Firstly, we constructed the primary model using structural equation model (SEM), and chronotype scores were chosen as independent variable and BMI values were taken as dependent variable. Secondly, we also constructed the reversed model using SEM, and BMI values were considered as independent variable and chronotype scores were regarded as independent variables. In two models, caffeinated drinks consumption, physical activity and inactivity time were considered as mediators to explore their effect for dependent and independent variable.

### 2.5. Statistical Analysis

Statistical analyses were conducted using SPSS 23 (SPSS Inc., Chicago, IL, USA), and mediational models were performed with SPSS PROCESS and Amos version 21. Prior to analysis, data was screened and checked to ensure accuracy. All 616 participants completed every item in this study. Missing data was handled using expectation maximization by using SPSS, and demographic variables were calculated through descriptive statistics, which were summarized via mean ± standard deviation (SD) or proportions (%). Moreover, Pearson correlation was conducted to explore the association between key study variables, including chronotype, BMI, and caffeinated drinks consumption. To identify the mediating effect of caffeinated drinks consumption and physical state on the association between chronotype and BMI, we employed the mediation model and bootstrap technique using SPSS PROCESS and Amos 21, respectively [31]. In addition, a total of three sociological variables or medical characteristics, namely, age, gender, and psychological distress total scores, were controlled in the model. Bootstrapping resampling procedure can reduce the likelihood of Type I error and modify non-normality of sampling distribution for small samples [32]. In this study, bootstrapping was used to estimate 10,000 resamples, and bias-corrected 95% confidence intervals (CIs) were used to evaluate the indirect effects. Supposing the (CIs) didn’t include the value 0, the indirect effect indicated significant existence.

## 3. Results

### 3.1. Participant Demographics Statistics and Correlates

The demographic characteristics of the current sample comprising 616 medical students (mean age 19.7 ± 1.1; 34.9% male) are displayed in Table 1. The result indicated that the average BMI (kg/m^2^) was 20.2 (SD 2.7) and caffeinated drinks consumption (L) was 0.6 (SD 0.4). Furthermore, respondents indicated that intermediate has an overall majority in chronotype (62.8%), followed by morningness (22.9%) and eveningness (14.3%). A total of 41% of participants (38.2%) were sophomore students who showed slight advantage in the sample distribution. In addition, most participants were apt to live on the campus (91.1%) than the people who lived on other places (8.9%). Finally, the mental and physical activity/inactivity states were reported in the study, and indicated that moderate physical activity time (h) and sedentary physical activity time (h) were 3.2 (SD 2.8) and 6.3 (SD 2.7), respectively. For the participants’ moderate/extremely psychological distress, anxiety become severe problems (19.6%), followed by depression (11.0%) and then stress (10.7%).

Table 2 displays the correlations between key study variables. Significant correlations were found between major research variables: BMI and the following variables, such as chronotype scores, caffeinated drinks consumption, physical activity time, and physical inactivity time. Interestingly, anxiety scores did not exhibit a statistically significant association between independent and outcome variables; however, a strong association was found between chronotype scores, caffeinated drinks consumption, BMI, and depression scores. Finally, a correlation between age and other key variables was not found.

### 3.2. Mediation Analyses

#### 3.2.1. Caffeinated Drinks Consumption as a Mediator in the Effect of Chronotype Scores on BMI

**Figure 1:**
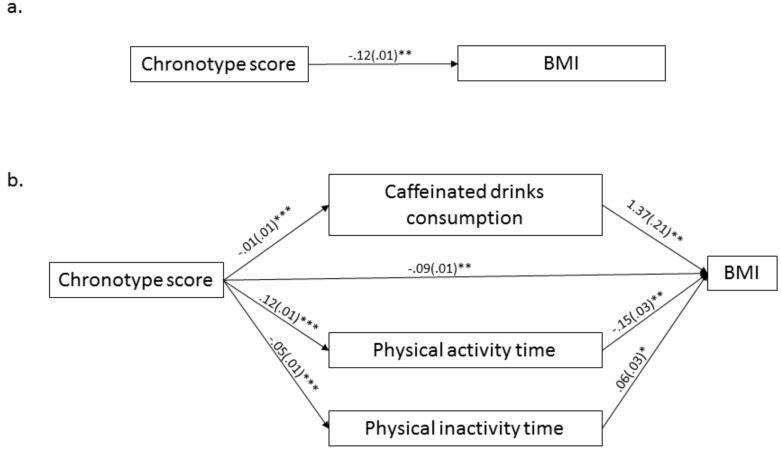
Primary multiple mediation model between chronotype scores and BMI values. Notes: (**a**) Direct effect relationship between chronotype scores and BMI; (**b**) Indirect effect relationship between chronotype scores and BMI in primary model. Bootstrapping coefficients are shown with standard error in the path diagram. * *p* < 0.05, ** *p* < 0.01, *** *p* < 0.001.

The results of the mediation model analysis are presented in Figure 1. Significant negative associations were observed among chronotype scores, caffeinated drinks consumption (B = −0.01, SE = 0.01, *p* < 0.001), and physical inactivity time (B = −0.05, SE = 0.01, *p* < 0.001). On the contrary, a positive relationship existed between chronotype scores and physical activity time (B = 0.12, SE = 0.01, *p* < 0.001). Hence, late chronotypes, such as eveningness, were associated with more caffeinated drinks intake, sedentary behavior and less physical activity time.

A significant positive association was found between assumed mediator of caffeinated drinks consumption (B = 1.37, SE = 0.21, *p* < 0.01), physical inactivity time (B = 0.06, SE = 0.03, *p* < 0.05), and the outcome of BMI. Moreover, a significant negative relationship was found between physical activity time and BMI (B = −0.15, SE = 0.01, *p* < 0.01). The result indicated that increased caffeinated drinks intake and physical inactivity time and reduced physical activity time are associated with high BMI. Although the association among physical activity time, chronotype scores, and BMI was significant, the 95% CI of the physical activity time was infinitely close to zero, and the mediating effect of physical activity time on BMI may be neglected in the results.

The relationship between high chronotype scores and low BMI was significant (B = −0.12, SE = 0.01, *p* < 0.001). After adjusting the indirect effects of the putative mediators, the relationship between independent and dependent variables remained significant but the effect weakened (B = −0.09, SE = 0.01, *p* < 0.01). Caffeinated drinks consumption partially significantly mediated the relationship between chronotype scores and BMI (estimate = −0.01, SE = 0.01, CI = (−0.02, −0.01). The overall model accounted for approximately 31% of the variance in BMI (R^2^ = 0.31, F = 68.69, *p* < 0.001).

#### 3.2.2. Caffeinated Drinks Consumption as a Mediator in the Effect of BMI on Chronotype Scores

**Figure 2:**
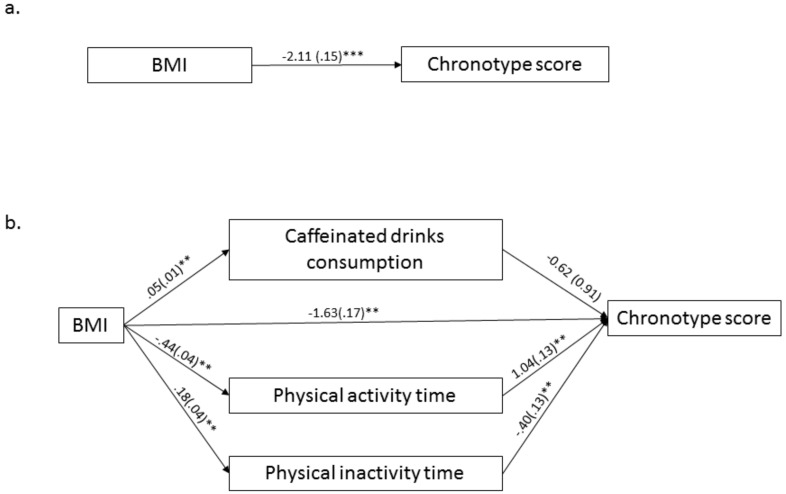
Reversed multiple mediation model between BMI values and chronotype scores. Notes: (**a**) Direct effect relationship between BMI and chronotype scores; (**b**) Indirect effect relationship between chronotype scores and BMI in reversed model. Bootstrapping coefficients are shown with standard error in the path diagram. ** *p* < 0.01, *** *p* < 0.001.

Reversed mediation analysis is presented in Figure 2. BMI had a positive significant association with increased caffeinated drinks consumption (B = 0.05, SE = 0.01, *p* < 0.01), high physical inactivity time (B = 0.18, SE = 0.04, *p* < 0.01), and reduced physical activity time (B = −0.44, SE = 0.04, *p* < 0.01).

The relationship between BMI and chronotype scores was significant (B = −2.11, SE = 0.15, *p* < 0.001). Hence, high BMI may be associated with low chronotype scores. However, after accounting for the indirect effects of the proposed mediators, the CIs for the indirect effect of caffeinated drinks consumption contained 0 (95% CI [−0.12, 0.06]), indicating that caffeinated drinks consumption did not significantly mediate the effect of BMI on chronotype scores.

A detailed summary of the results from both mediation models is provided in Table 3.

## 4. Discussion

The purpose of this survey was to explore whether caffeinated drinks consumption is a potential mediator between the association with chronotype and BMI among medical undergraduates. A bidirectional association between chronotype and BMI has been established [15,19,33,34]. However, most studies did not focus on clarifying the influence path between independent and dependent variables through other key variables in relevant research, and their underlying effect mechanisms were overlooked. Our study indicated that caffeinated drinks consumption could mediate the effect of chronotype scores on BMI. However, caffeinated drinks consumption was insignificant in the effects of BMI on chronotype scores. The above results found that a high intake of caffeinated drinks may be an important pathway in which high chronotype scores confers with high-risk BMI values.

When the analyses were conducted by primary and reverse mediation models, the pattern of results showed a few different effects between chronotype scores and BMI, respectively. A significant total high chronotype scores and low BMI, which were partially mediated by more caffeinated drinks consumption, were observed in the primary mediation model. This result is consistent with previous studies that indicated that stimulant drinks may be a crucial factor between a late chronotype and increased BMI [35,36]. Giannotti et al. reported that in response to the adverse effect of daytime sleepiness, eveningness subjects required more stimulants, such as caffeine and caffeine-containing beverages [36]. Meanwhile, this phenomenon was easily observed in follow-up studies by food logs for seven days [35]. Additionally, Martin et al. found that eveningness subjects reported high-levels of chronic work-related fatigue [37], which can aggravate the intake of caffeinated drinks. Interestingly, in reverse mediation, no significant mediation association was found between BMI and chronotype scores through caffeinated drinks intake. This finding can be attributed to two reasonable explanations: one is that chronotype development is determined by other internal factors, such as gene, age, sex, and environment, except for individual behavior [38]. Moreover, caffeinated drinks consumption only belongs to one kind of stimulation; other substances, such as tobacco and alcohol, have also been reported to be positively associated with late chronotype [39]. Another reason is that the multi-mediation model showed a strong association between physical activity time, physical inactivity time, and chronotype scores in this cross-sectional study, thus weakening the effect in the model. Therefore, further cohort studies should be considered to clarify the mediation effect of caffeinated drinks consumption between BMI and chronotype scores. Finally, we found that caffeinated drinks consumption was not indirectly associated with BMI and chronotype scores in the reverse model; however, the results suggested that BMI was directly related with chronotype scores. This is a novel approach that was rarely reported in previous studies.

These results found that late chronotype is associated with the risk of being overweight and through caffeinated drinks consumption. Although it cannot illuminate a causal relationship because the study is based on a cross-sectional survey, a late chronotype can potentially contribute to increased BMI via an augmentation in caffeinated drinks intake. In turn, a high caffeinated drinks intake promotes high BMI and obesity via a series of possible mechanisms. First, sugar-sweetened beverages and coffee often follow a high dose of caffeine to improve the flavor of drinks as an addictive psychoactive chemical. Therefore, individuals who continue intake of these drinks may increase their addiction opportunity to these drinks, which can contribute to an increase in sugar intake and may influence weight gain [40]. This finding has received support in randomized controlled trials, and was further considered “the caffeine-calorie effect”, which means that the caffeine in sugar beverages has a small effect on unhealthy weight gain for the average individual but could be observed in large populations [41]. Second, a high caffeinated drinks intake can affect the physiological regulation of the human body. For example, a single serving of coffee considerably heightens the postprandial glycemic response in healthy overweight men [42], which can support a potential mechanism explanation for the effects of instant coffee. Furthermore, caffeine consumption in some levels may result in improved insulin sensitivity, which can play an important role in energy regulation and expenditure [43], and obesity is always associated with insulin resistance and type 2 diabetes [44]. Meanwhile, late chronotype may aggravate the chance to consume caffeinated drinks. First, late chronotypes has been found to be prone to developing the misalignment of internal and external time and accumulate considerable sleep debt during work days [39]. Daytime napping may be increased as a response to this condition and offset inefficient sleep. However, the majority of academic courses are during the daytime; thus, students are compelled to intake more stimulation, and caffeinated drinks are a highly common choice. Second, medical students bear rigorous academic and clinical training, easily resulting in sleep disturbances, perceived academic stress, and depression [45,46]. In a meta-analysis, researchers found that the dose-response studies between coffee consumption and the risk of depression confirmed a J-shaped tendency, and proper coffee consumption may decline the low risk of depression among populations [47]. Furthermore, this finding potentially causes the late chronotype consumption of additional caffeinated drinks among individuals in high-stress jobs.

Aside from chronotype, many other potential pathways are also available to mediate the effect between late chronotype and increased BMI. For example, participants who belong to late chronotype may feel increased exhaustion during the daytime, leading them to shorter engagement in physical activity and increased time in sedentary states [48,49]. The above research hypothesis was further supported in this study. Interestingly, we found that the effect of BMI on physical activity and inactivity times was significant. This finding could reveal another possible assumption, which indicates that the link among sedentary behavior, physical activity and BMI was not only highly correlated [50]. In other words, their relationship may not be a one-way process but may be a mutual cause-and-effect relationship. Under this mechanism, vicious or healthy circles may be generated. For example, an overweight person must pay increased effort and perseverance during exercise, which may result in reduced enthusiasm for performing physical activities. Moreover, the effect of physical inactivity time mediating the relationship between chronotype scores and BMI is weak in the preliminary model. The explanation may be that the strong significant association between chronotype scores and physical inactivity time led to path weakening. In addition, the Hawthorne effect may exist in data collection between weight and physical inactivity. Moreover, the effect of physical activity and inactivity time on the chronotype scores was notable. In previous studies, light exposure was considered an important zeitgeber in circadian systems [51], which can affect melatonin secretion and extend the entrainment phase, thereby developing late chronotypes [52,53]. In the university, students will spend more time staying indoors (such as classrooms, and dorms) than outdoors, and they generally experience a zeitgeber reduction because they are exposed to less light during the daytime, and the people with additional physical activities have a considerably higher possibility of light exposure. This finding may provide a key explanation for their relationship. However, further causality requires additional sample support and multi-latitudinal research.

Several limitations of this study should be addressed. First, this study used a self-reporting questionnaire, which can be imprecise, particularly during information collection. Therefore, further research should utilize objective measures to collect physical activity/inactivity time, drinks consumption, and BMI. Second, gender difference should focus on an increased sample study; we did not find the difference in univariate analysis for diverse gender possibly due to the restricted sample size. Third, the data from the cross-sectional study did not allow for confirmation of the direction of causation. For example, although late chronotype contributes may contribute to increase BMI, a high BMI may contribute to late chronotype via other unhealthy eating habits, such as high-salt and high-fat diet. Finally, the age distribution is limited in view of the research design, and further research on this topic can explore the different associations among youth, middle age, and old age. Hence, further longitudinal studies should be conducted.

## 5. Conclusions

This study found that caffeinated drinks consumption might mediate the relationship between late chronotype and increased BMI among medical students. This finding can provide possible new evidence to enrich the effect of the eating behavioral model of BMI to the circadian rhythms theory. This theory may suggest that school schedule should be reasonably arranged and the intake of stimulants may be reduced. In addition, physical activity and sedentary behavior can be key mediators in this model, which should be in equilibrium to cultivate improved sleep and biological rhythms and interact with low weight to maintain health. However, our research data was collected through a cross-sectional survey and only 616 participants were included in this study. This result did not permit us to confirm the causality. Therefore, if the research can be repeated in a large study, then relevant precaution and education may be warranted. In spite of these limitations, this study used SEM model to provide implications for interventions that could be further designed for reducing caffeinated drinks consumption and increased physical activity time to maintain the weight balance.

## Figures and Tables

**Table 1:** Demographic Characteristics for the Total Sample (*n* = 616).

Characteristic	Numbers (%)
	Mean ± SD
Age (years)	19.7 ± 1.1
Gender, *n* (%)	
Male	215 (34.9%)
Female	401 (65.1%)
BMI (kg/m^2^)	20.2 ± 2.7
Residence, *n* (%)	
Campus	561 (91.1%)
Others	55 (8.9%)
Grade, *n *(%)	
Freshman	191 (31.0%)
Sophomore	253 (41.1%)
Junior	172 (27.9%)
Moderate Physical Time (hours)	3.2 ± 2.8
Sedentary Physical Time (hours)	6.3 ± 2.7
Chronotype, *n* (%)	
Morningness	141 (22.9%)
Intermediate	387 (62.8%)
Eveningness	88 (14.3%)
Caffeinated Drinks Consumption (L)	0.6 ± 0.4
DASS-C21 Scale	
Total scores	31.7 ± 5.1
Depression (moderate/extremely), *n* (%)	68 (11.0%)
Anxiety (moderate/extremely), *n* (%)	121 (19.6%)
Stress (moderate/extremely), *n* (%)	66 (10.7%)

**Table 2:** Correlations of Key Study Variables.

	1	2	3	4	5	6	7	8	9
1. Chronotype	1								
2. CDC	−0.20 **	1							
3. BMI	−0.51 **	0.31 **	1						
4. Age	−0.04	−0.03	0.06	1					
5. PAT	0.44 **	−0.18 **	−0.38 **	−0.03	1				
6. PIT	−0.20 **	0.05	0.18 **	0.02	−0.10 *	1			
7. Depression	−0.13 **	0.13 **	0.14 **	−0.07	0.02	0.03	1		
8. Anxiety	0.03	−0.02	−0.02	−0.05	0.01	−0.01	0.01 **	1	
9. Stress	0.20 **	−0.03	−0.36 **	−0.01	0.05	−0.11 **	−0.30 **	−0.20	1

Notes: CDC = Caffeinated Drinks Consumption; BMI = Body Mass Index; PAT = Physical Activity Time; PIT = Physical Inactivity Time. * *p* < 0.05; ** *p* < 0.01.

**Table 3:** Bootstrapping analyses were used in primary and reversed model, respectively.

Variables	B	SE	95% BC CI	*p*
Model 1 (outcome: BMI)				
Effect of predicator on outcome				
Chronotype scores → BMI	−0.12	0.01	(−0.13, −0.10)	<0.001
Effect of predicator on mediators				
Chronotype scores → CDC	−0.01	0.01	(−0.01, −0.00)	<0.001
Chronotype scores → PAT	0.12	0.01	(0.10, 0.14)	<0.001
Chronotype scores → PIT	−0.05	0.01	(−0.07, −0.03)	<0.001
Effect of mediators on outcome				
CDC → BMI	1.37	0.21	(0.96, 1.78)	<0.01
PAT → BMI	−0.15	0.03	(−0.21, −0.08)	<0.01
PIT → BMI	0.06	0.03	(0.03, 0.19)	0.05
Model 2 (outcome: Chronotype)				
Effect of predicator on outcome				
BMI → Chronotype	−2.11	0.15	(−2.40, −1.81)	<0.001
Effect of predicator on mediators				
BMI → CDC	0.05	0.01	(0.04, 0.06)	<0.01
BMI → PAT	−0.44	0.04	(−0.52, −0.35)	<0.01
BMI → PIT	0.18	0.04	(0.10, 0.27)	<0.01
Effect of mediators on outcome				
CDC → Chronotype scores	−0.62	0.91	(−2.42, 1.17)	0.50
PAT → Chronotype scores	1.04	0.13	(0.78, 1.30)	<0.01
PIT → Chronotype scores	−0.40	0.13	(−0.65, −0.14)	<0.01
**Indirect Effects**	**Estimate**	**SE**	**LL 95% CI**	**UL 95% CI**
Model 1 (outcome: BMI)				
CDC	−0.01	0.01	−0.02	−0.01
PAT	−0.02	0.01	−0.04	−0.01
PIT	−0.01	0.01	−0.01	0.004
Model 2 (outcome: Chronotype)				
CDC	−0.03	0.05	−0.12	0.06
PAT	−0.45	0.09	−0.65	−0.30
PIT	−0.07	0.03	−0.15	−0.03

Notes: (a) BMI = body mass index; EDS = Caffeinated drinks consumption; PAT = physical activity time; PIT = physical inactivity time. (b) SE = standard error; 95% BC CI = 95% bias-corrected confidence intervals. LL = lower level; UL = upper level.
